# Supplementary material for: Perceived impacts of COVID-19 and bushfires on the implementation of an obesity prevention trial in Northeast Victoria, Australia
Source: PLoS One. 2023 Jun 20;18(6):e0287468. doi: 10.1371/journal.pone.0287468 (PMC10281563; doi:10.1371/journal.pone.0287468)
Supplement: S1 File — (PDF) [file pone.0287468.s001.pdf]

Focus Group Facilitator Guide/Interview Schedule

Focus group facilitators will use the Durlak and DuPre Framework below to guide stakeholders through the barriers and enablers to effective implementation previously identified in the literature.

Once the conversation commences, it is envisaged that each specific dot point noted below will not need to be worked through in a step-by-step fashion. One facilitator and one note-takers will be present at each of the focus groups to ensure maximum information is recorded from the conversations

In the schedule provided below, a question is posed, the dotpoints below each question will be used if clarification is required to prompt discussion.

START

Introduction:

Thank you for agreeing to come to this focus group discussion today on the impacts of COVID19 and other disruptions, such as bushfires and staff turnover on the prevention work that you are involved with. I'll just briefly re-introduce myself in case some of you don't know me. My name is Jill Whelan, I have now been at Deakin University for ten years, prior to that I worked in food insecurity in the homeless youth sector and was a teacher for 17 years before that. I enjoy working with communities to understand the change process, particularly in relation to healthy eating and active living. We also have Monique here from Deakin, Monique will be taking notes of our discussion today just in case technology lets us down.

Firstly, I want to make it clear that this is part of the RESPOND research, where we will undertake focus groups with each of the RESPOND communities and cover these same discussion points. If you wish to participate, we do need to return your signed informed consent form. I'd like to pause here to check if there are any signed forms outstanding.

If there are signed forms outstanding, I will read the PLS and ask people to write into the chat function their name and consent and to forward the PLS to us so we are able to analyse and use this data. If they choose not to consent, they are welcome to either leave the call or just listen and not participate in the discussion.

Recording on now:

There are a few different themes that I will introduce into the discussion today, around community factors, workplaces, work roles, funding, policy etc and ask for your reflections as you think back to what has impacted your work role in RESPOND since either you (or others) ran the GMB 3 workshop for this community of {Euroa and Strathbogie Shire}. If you have joined the RESPOND workforce since then, your insights are also extremely valuable.

We (Deakin) understand that RESPOND has progressed in different ways in different areas, and it is important for us to formally document how COVID and bushfires and other things have impacted RESPOND. We hope to publish the work from these focus groups, and we may use quotes of things that you say during this focus group within the manuscript. However the information across all of the focus groups will be summarised and no individual person will be identified.

Our aim today is to have a relaxed and open conversation about the last almost two years of working around COVID, bushfires and other impacts on RESPOND and your work. We will transcribe the

recording of this conversation and will send back to you a copy of the transcript to approve before we analyse it.

1. **I am going to start with the first point of discussion which is about your experience of working with your community. RESPOND, as we all know, is based on the concept of co-design and shared decision making with local community stakeholders, local leaders, local experts with a bit of academic advice included**. *(IVB)*

   In what ways do you or did you experience or not experience this sense of shared decision-making with your community, for example, the GMBs, or working groups?
   - *Prompts:*
     - *Coming to the GMBs*
     - *Implementing ideas*
     - *Forming partnerships*
     - *What does the collaboration and cooperation between local agencies look like?*

2. The second discussion point carries on with communities but also includes your various workplaces and work roles: ***(for analysis item II: Provider Characteristics A, B, C, D)***

- **I'd like to hear from you if you consider that your workplaces feel they are able to do what they think is expected from them for RESPOND?**

  - Possible prompts:
    - *To what extent do you perceive BOTH your workplaces and YOUR COMMUNITY considers that RESPOND is* ***relevant*** *to the local community? ITEM II provider characteristics AND item IV part B1,*
    - *Do you consider your workplaces thinks that RESPOND* ***can achieve benefits*** *to children's health – why/why not?*

    - ***In your experience so far, are there enough people and organisations with the relevant skills to implement RESPOND?***

Thank you! I am really interested to hear what is happening in your workplaces and work roles in what has just been an incredibly strange time for so many people.

Follow up point: Is there something specific that you can see would help you to move through these issues?

Thank you, these are really valuable insights, thanks so much for sharing.

3. **Since RESPOND started, it seems that you have experienced lots of changes in leadership, local champions, managerial support etc., Please tell us a little about what has happened without using 'names', and how this has impacted RESPOND.**
   - Leadership
   - Program champion (internal advocate)
   - Managerial/supervisory/administrative support

- **What do you need to move forward with this work?**

4. **Do you consider that you and other staff in RESPOND have received enough training in systems thinking, STICKE and in implementation** to conduct RESPOND.

- What else do you need?

5. Thinking about BIG 'community level' factors, can you give us some examples of things that have influenced the implementation of RESPOND so far, these may be funding, policies, it could be politics, anyone like to start?.... *(for analysis: item I. community level factors: prevention theory and research, politics, funding, policy)*

   Let conversation flow:

   - Possible prompt:
     - has local attitudes to how 'prevention' is prioritised impacted your work in any way.
     - In your experience how well does your community accept/enjoy/adopt change or something new?

6. We're nearly finished here, I'd like you to reflect further specifically on **RESPOND as you have experienced it:**

- Has RESPOND been a good fit with your organisation and your community?
- Have you felt empowered to make any changes that you needed or wanted to do to make it work for your workplace and/or your community?

7. If you were starting again, what would you differently?

Durlak JA, DuPre EP. Implementation matters: a review of research on the influence of implementation on program outcomes and the factors affecting implementation. Am J Community Psychol. 2008 Jun;41(3-4):327-50. doi: 10.1007/s10464-008-9165-0. PMID: 18322790.
