# Supplementary material for: Perceived impacts of COVID-19 and bushfires on the implementation of an obesity prevention trial in Northeast Victoria, Australia
Source: PLoS One. 2023 Jun 20;18(6):e0287468. doi: 10.1371/journal.pone.0287468 (PMC10281563; doi:10.1371/journal.pone.0287468)
Supplement: S2 File — (PDF) [file pone.0287468.s002.pdf]

Poll Questions:

Please rate each of these statements from strongly disagree to strongly agree.

1. My workplace considers RESPOND relevant to local needs.
   - ☐ Strongly disagree
   - ☐ Disagree
   - ☐ Neutral
   - ☐ Agree
   - ☐ Strongly agree

2. My community considers RESPOND relevant to local needs.
   - ☐ Strongly disagree
   - ☐ Disagree
   - ☐ Neutral
   - ☐ Agree
   - ☐ Strongly agree

3. Our organisation has enough funding/can re-orient funding to meet the needs of RESPOND.
   - ☐ Strongly disagree
   - ☐ Disagree
   - ☐ Neutral
   - ☐ Agree
   - ☐ Strongly agree

4 The prevention theory and research component of RESPOND fits our community priorities and way of working.
   - ☐ Strongly disagree
   - ☐ Disagree
   - ☐ Neutral
   - ☐ Agree
   - ☐ Strongly agree

5. Local politics (mayor/council/local govt reps) are supportive of RESPOND.
   - ☐ Strongly disagree
   - ☐ Disagree
   - ☐ Neutral
   - ☐ Agree
   - ☐ Strongly agree

6. Our workplace/team members have the skills needed to run group model building.
   - ☐ Strongly disagree
   - ☐ Disagree
   - ☐ Neutral
   - ☐ Agree
   - ☐ Strongly agree

7. Our workplace/team members have the skills and connections needed to implement RESPOND actions.
   - ☐ Strongly disagree
   - ☐ Disagree
   - ☐ Neutral
   - ☐ Agree
   - ☐ Strongly agree

8. State policy supports the approach of RESPOND.
   - ☐ Strongly disagree
   - ☐ Disagree
   - ☐ Neutral
   - ☐ Agree
   - ☐ Strongly agree

9. There is a positive work climate in our organisation. Morale, trust, collegiality and dispute resolution is strong.
   - ☐ Strongly disagree
   - ☐ Disagree
   - ☐ Neutral
   - ☐ Agree
   - ☐ Strongly agree

10. RESPOND is adaptable to the needs of our workplace.
    - ☐ Strongly disagree
    - ☐ Disagree
    - ☐ Neutral
    - ☐ Agree
    - ☐ Strongly agree

11. RESPOND is adaptable to the needs of our community.
    - ☐ Strongly disagree
    - ☐ Disagree
    - ☐ Neutral
    - ☐ Agree
    - ☐ Strongly agree

12. We/our workplace(s) have a strong commitment to a shared vision that aligns with the vision of RESPOND i.e. to co-create community-led change to improve the health of children.
    - ☐ Strongly disagree
    - ☐ Disagree
    - ☐ Neutral
    - ☐ Agree
    - ☐ Strongly agree

13. Our community partnerships are strong.
   - ☐ Strongly disagree
   - ☐ Disagree
   - ☐ Neutral
   - ☐ Agree
   - ☐ Strongly agree

14. We have strong strategic planning, clear roles and responsibilities within RESPOND.
   - ☐ Strongly disagree
   - ☐ Disagree
   - ☐ Neutral
   - ☐ Agree
   - ☐ Strongly agree

15. We have adequate access to technical assistance, training, skills development within RESPOND.
   - ☐ Strongly disagree
   - ☐ Disagree
   - ☐ Neutral
   - ☐ Agree
   - ☐ Strongly agree
