# Supplementary material for: Perceived impacts of COVID-19 and bushfires on the implementation of an obesity prevention trial in Northeast Victoria, Australia
Source: PLoS One. 2023 Jun 20;18(6):e0287468. doi: 10.1371/journal.pone.0287468 (PMC10281563; doi:10.1371/journal.pone.0287468)
Supplement: S1 Table — (PDF) [file pone.0287468.s003.pdf]

RESPOND Pause and Reflect Codebook

Items in *italics* are not from Durlak et al. table 2. They have been added either as new themes by the researchers, or definitions from within the text of Durlak et al.

Major Theme	Definition	Minor theme	Definition	Mini theme	Definition
1. Community level factors		Prevention Theory and Research	*Include dissemination.*	*Readiness*	
		Politics	*Can be positive or negative*		
		Funding	*Can be positive or negative*	*the 'right' people*	
		Policy	*New policy and procedures and also support through admin / financial infrastructure*		
2. Provider Characteristics		Perceived Need for Innovation	Extent to which the proposed innovation is relevant to local needs		
		Perceived Benefits of Innovation	Extent to which the innovation will achieve benefits desired at the local level		
		Self-efficacy	Extent to which providers feel they are / will be able to do what is expected		
		Skill Proficiency	Possession of the skills necessary for implementation		
3. Characteristics of the Innovation		Compatibility (contextual appropriateness, fit, congruence, match)	Extent to which the intervention fits with an organization's mission, priorities, and values.		
		Adaptability (program modification, reinvention)	The extent to which the proposed program can be modified to fit provider preferences, organizational		

Major Theme	Definition	Minor theme	Definition	Mini theme	Definition
			practices, and community needs, values, and cultural norms. *Flexibility.*		
		Co-design			
		Community engagement			
		Momentum			
4. Factors Relevant to the Prevention Delivery System: Organizational Capacity		General Organizational Factors		Positive Work Climate	Climate may be assessed by sampling employees' views about morale, trust, collegiality, and methods of resolving disagreements
				Organizational norms regarding change (a k a, openness to change, innovativeness, risk-taking)	This refers to the collective reputation and norms held by an organization in relation to its willingness to try new approaches as opposed to maintaining the status quo
				Integration of new programming	This refers to the extent to which an organization can incorporate an innovation into its existing practices and routines
				Shared vision (shared mission, consensus, commitment, staff buy-in)	This refers to the extent to which organizational

Major Theme	Definition	Minor theme	Definition	Mini theme	Definition
					members are united regarding the value and purpose of the innovation
		Specific Practices and Processes		Shared decision-making (local input, community participation or involvement, local ownership, collaboration)	The extent to which relevant parties (e.g., providers, administrators, researchers, and community members) collaborate in determining what will be implemented and how. *Also predicts program sustainability.*
				Coordination with other agencies (partnerships, networking, intersector alliances, multidisciplinary linkages)	The extent to which there is cooperation and collaboration among local agencies that can bring different perspectives, skills, and resources to bear on program implementation
				Communication	Effective mechanisms encouraging frequent and open communication
				Formulation of tasks (workgroups, teams, formalization, internal functioning, effective human resource management)	Procedures that enhance strategic planning and contain clear roles and

Major Theme	Definition	Minor theme	Definition	Mini theme	Definition
					responsibilities relative to task accomplishments
		Specific Staffing Considerations		Leadership	Leadership is important in many respects, for example, in terms of setting priorities, establishing consensus, offering incentives, and managing the overall process of implementation
				Program champion (internal advocate)	An individual who is trusted and respected by staff and administrators, and who can rally and maintain support for the innovation, and negotiate solutions to problems that develop
				Managerial/supervisory/administrative support	Extent to which top management and immediate supervisors clearly support and encourage providers during implementation
				Staff turnover (added)	*Wherever a participant mentions staff churn/turnover/staff changes etc*
				Staff time (Added)	

Major Theme	Definition	Minor theme	Definition	Mini theme	Definition
				Reliance on volunteers (added)	*(The framework assumes paid workforce, we need to make it clear that in our work paid workforce relies on community/volunteer support*
5. Factors Related to the Prevention Support System		Training	Approaches to ensure provider proficiencies in the skills necessary to conduct the intervention and to enhance providers' sense of self efficacy. *Goal is to prepare providers effectively for their new tasks. Also attend to their expectations, motivation.*		
		Technical Assistance	This refers to the combination of resources offered to providers once implementation begins, and may include retraining in certain skills, training of new staff, emotional support, and mechanisms to promote local problem-solving efforts. *RE-training of initial providers.*		
6. *Environmental Shocks*					
