# Supplementary material for: Perceived impacts of COVID-19 and bushfires on the implementation of an obesity prevention trial in Northeast Victoria, Australia
Source: PLoS One. 2023 Jun 20;18(6):e0287468. doi: 10.1371/journal.pone.0287468 (PMC10281563; doi:10.1371/journal.pone.0287468)
Supplement: Attachment — Submitted filename: Response to Reviewers.docx [file pone.0287468.s005.docx]

RESPONSE TO REVIEWERS

| **Comment by Reviewer** | **Response** | **Reference/ page & line number (final)** |
| --- | --- | --- |
| 1. Please ensure that your manuscript meets PLOS ONE's style requirements, including those for file naming. | Formatting changes have been made to the file names, cover page, headings, referencing and citations | All pages |
| 2. Please provide additional details regarding participant consent. Please ensure that you have specified (1) whether consent was informed and (2) what type you obtained (for instance, written or verbal, and if verbal, how it was documented and witnessed).   Once you have amended this/these statement(s) in the Methods section of the manuscript, please add the same text to the “Ethics Statement” field of the submission form (via “Edit Submission”). | Information about consent has been provided in the Methods section of the manuscript. | Line 130 - 133  Ethics Statement in submission form |
| 3. Please state what role the funders took in the study.  If the funders had no role, please state: "The funders had no role in study design, data collection and analysis, decision to publish, or preparation of the manuscript."  Please include this amended Role of Funder statement in your cover letter; we will change the online submission form on your behalf. | Information about the funders and their role in the manuscript has been provided in the cover letter. | Cover letter |
| 4. Please complete your Competing Interests on the online submission form to state any Competing Interests. If you have no competing interests, please state "The authors have declared that no competing interests exist.", as detailed online in our guide for authors at <http://journals.plos.org/plosone/s/submit-now>  This information should be included in your cover letter; we will change the online submission form on your behalf. | Information about competing interests has been included in the cover letter. | Cover letter |
| 5. Upon re-submitting your revised manuscript, please upload your study’s minimal underlying data set as either Supporting Information files or to a stable, public repository and include the relevant URLs, DOIs, or accession numbers within your revised cover letter. For a list of acceptable repositories, please see <http://journals.plos.org/plosone/s/data-availability#loc-recommended-repositories>. Any potentially identifying patient information must be fully anonymized.   Important: If there are ethical or legal restrictions to sharing your data publicly, please explain these restrictions in detail. Please see our guidelines for more information on what we consider unacceptable restrictions to publicly sharing data: <http://journals.plos.org/plosone/s/data-availability#loc-unacceptable-data-access-restrictions>. Note that it is not acceptable for the authors to be the sole named individuals responsible for ensuring data access.  We will update your Data Availability statement to reflect the information you provide in your cover letter. | The data in this study were collected by the study authors for the purposes of this study. The data were collected from a small rural town which is part of a larger funded trial. If the data were made available, participants would be potentially identifiable and such potential identification is not permitted under our ethics approval obtained from Deakin University HEAG-H 172_2018. | Explanation and alternate contact details provided on cover letter and submission portal |
| 6. Please include captions for your Supporting Information files at the end of your manuscript, and update any in-text citations to match accordingly. Please see our Supporting Information guidelines for more information: <http://journals.plos.org/plosone/s/supporting-information>. | The appendices have been reworded as Supporting Information files and cited in the relevant section of the manuscript. | S1_File – cited line 136  S1_Table – cited line 164 and 172 |
| Additional Comments  The reviewers comment that your manuscript could be strengthened with additional detail and clarification, as well as a refocus of the introduction so that it includes more detail on the activities involved in the RESPOND intervention.  In addition, one of the reviewers states that some of the conclusions drawn do not seem to stem from the data gathered, or at least reported in this study. | The explanation of RESPOND has been enhanced, along with the provision of more tangible examples of activities undertaken as part of the project.  The conclusion section has been tidied up to provide a stronger conclusion of the study. | Line 70 – 83  Line 540 - 555 |
| **REVIEWER 1** | | |
| This paper discusses the implementation experience of study team members in the context of bushfires and COVID-19. Overall, the paper would benefit from more clear and concise writing, particularly the introduction and the discussion. | The introduction and discussion have had significant edits and we believe read much more clearly and concisely. | All |
| Title 1. I recommend changing the title to something that more specifically describes the qualitative nature of the study. Perhaps “Perceived impacts of…” or “Facilitators and barriers to implementation of…” | The title has been adjusted appropriately. | Line 3 |
| Introduction 1. Overall, I find the introduction to be too long, with excessive background on the problem of obesity and bushfires, and not enough background on what activities are actually involved in the RESPOND intervention. It would be helpful to have a figure or table that lays out more detailed examples of projects/prioritized actions taken by some of the community stakeholders in their communities. The description as it is, remains very abstract and it is difficult to get a full sense of their work within RESPOND. | The background of obesity and bushfires has been shortened.  The explanation of RESPOND has been enhanced, along with the provision of more tangible examples of activities undertaken as part of the project. | Line 50 - 99 |
| Methods 1. Line 115: what is a Step 1 community? 2. Line 160: There should be a reference to Figure 1 here. | 1. The explanation of a step 1 and 2 community has been provided in the introduction about RESPOND. 2. Due to the formatting requirements of PLOS ONE, we are unable to reference the figure until just before the figure is provided in the manuscript. | Line 73 – 75  Line 174 and Line 473 |
| Results 1. Line 180-206: I find it difficult to follow this paragraph. Who is issuing the proposed policy changes that were provided to local stakeholders for review? What were some of these policy changes? What are the “new policy guidelines” that the participant is referring to as the big elephant in the room? 2. Line 304: What is LGA? 3. Line 434: This portion of the paper would benefit from a sentence or two to help with transition to the next section, rather than just ending on a quote. | 1. The wording of this section has been altered to make the meaning more clear. 2. All LGA acronyms have been spelt out. 3. This statement has been added: “These qualitative findings are expanded through the inclusion of the survey data, that provided more detail to some of the themes.” | Line 201 – 207  Line 316-318 and line 415  Line 448 - 449 |
| Discussion 1. Line 476-480: This particular conclusion does not seem to stem from the data gathered. Did some participants discuss policy changes that seemed to occur rapidly with political will? 2. Lines 481-487: The two examples given to cite benefits of online engagement are very specific and seem quite unrelated to the work that the RESPOND team was trying to do online. Are there more relevant examples in the literature? 3. Lines 505-end: All of the text after the conclusion reads like an unfinished outline, rather than the end of a scientific manuscript. Perhaps there is a PLOS ONE style that I am not familiar with but I find it difficult to follow.. | 1. This has been reworded so as not to indicate that political will has been observed from this particular study. 2. This paragraph has been reworded and more applicable references sourced and cited. 3. Some sections under the conclusion have been moved to a more appropriate location within the manuscript:    a. ‘Strengths & weaknesses’ to Discussion    b. Figure 1 to Results   Sub- headings under the Conclusion have been removed. | Line 504 – 507  Line 508 – 512  Line 540 - 555 |
| Table 1 1. I recommend removing the “N” from each table cell and just noting “n(%)” at the top of the table to indicate what format the data is presented in. | Table has been adjusted appropriately. | Page 21-22 |
| Figure 1 1. The figure is blurry and contains a lot of information that is not explained. It should have a legend that walks the reader through each piece of the figure and how to interpret what it is representing. | A more detailed explanation of the figure has been provided in the manuscript.  A higher resolution image has been provided and in a file type requested in the formatting guidelines. This image has also been processed through PACE. | Line 462 – 472  Figure 1 attachment |
| **REVIEWER 2** | | |
| Great work - this is a very important and unique topic. I have not thought about how bushfires might impact upon treatment for obesity before. Given Australia's obesity epidemic and the sheer number of people that the bushfires impacted upon, this is likely a topic that affects many people. I thought that your discussion around online support for obesity not being well liked (as opposed to online treatment for Diabetes) to be particularly useful for clinicians who design interventions for people who are obese. The methods of the article appear sound. There are some minor language errors, please do a further proofread of the article. | The authors thank the reviewer for acknowledging this unique insight.  The manuscript has had a final proofread before being resubmitted. | All |
